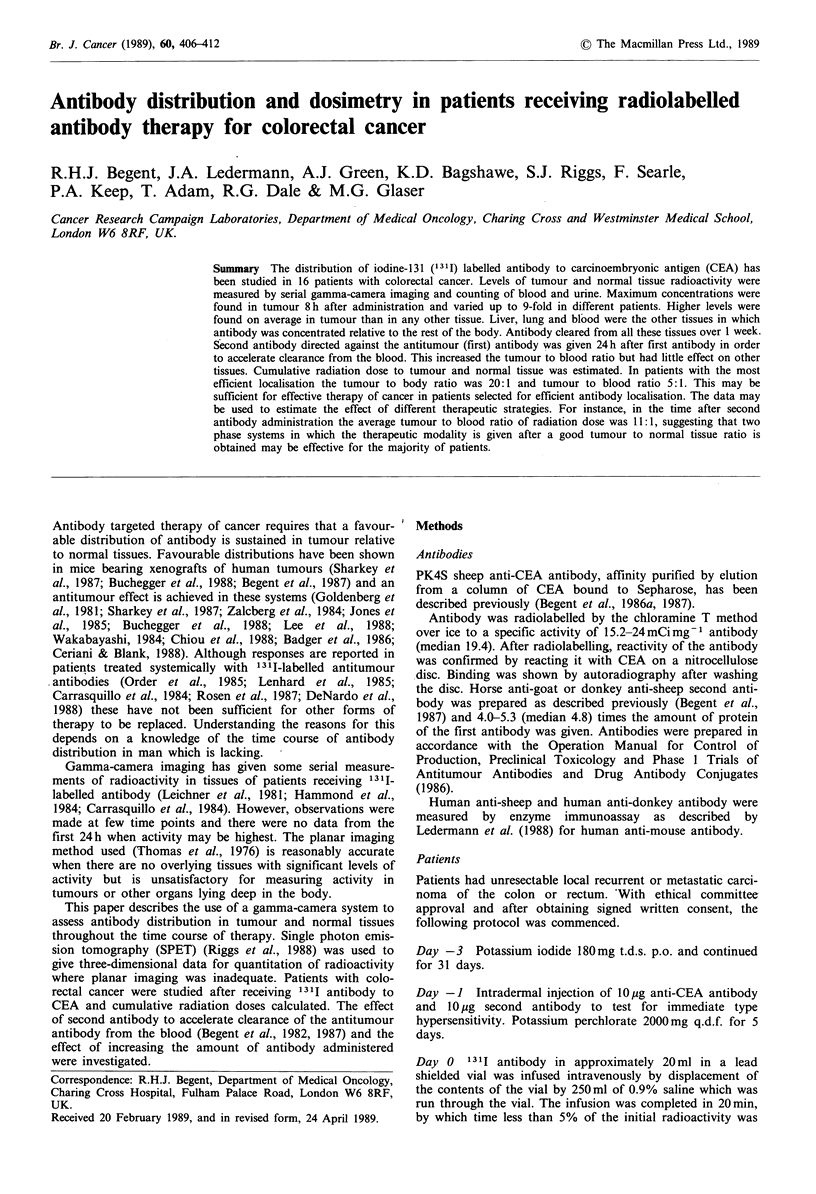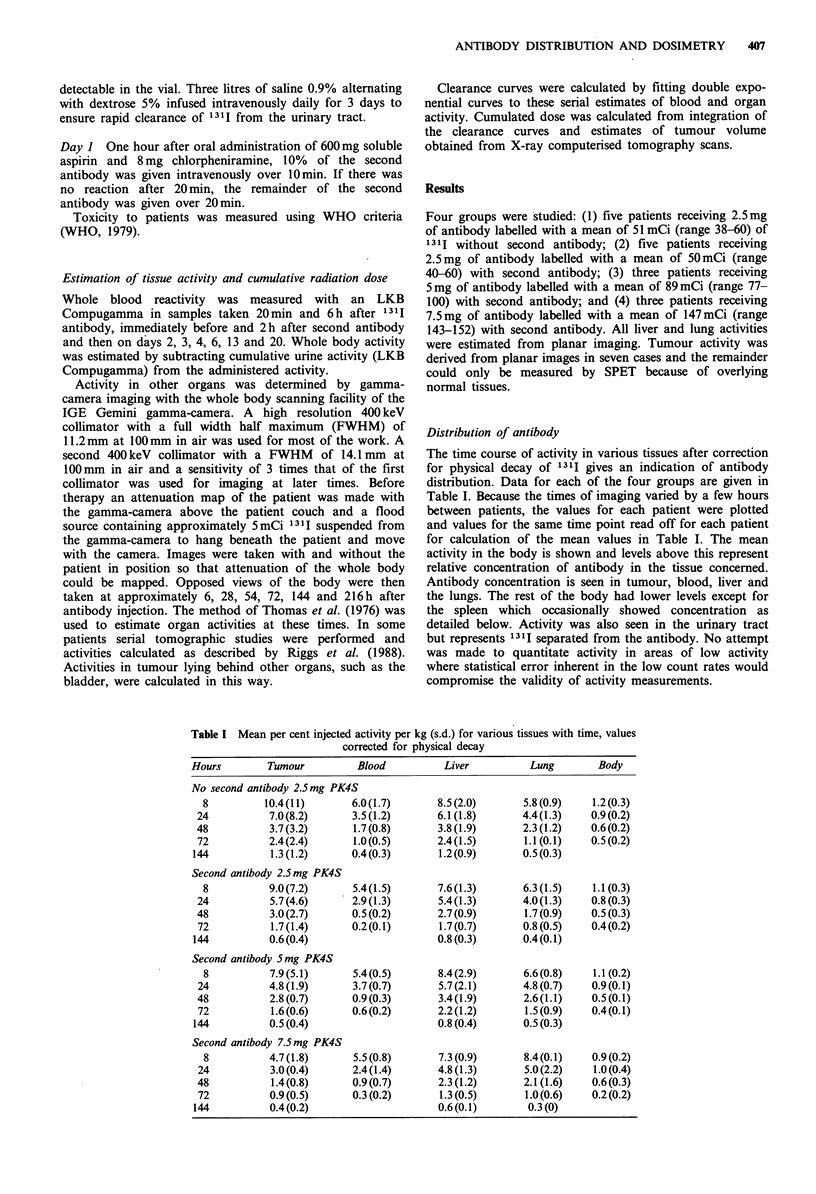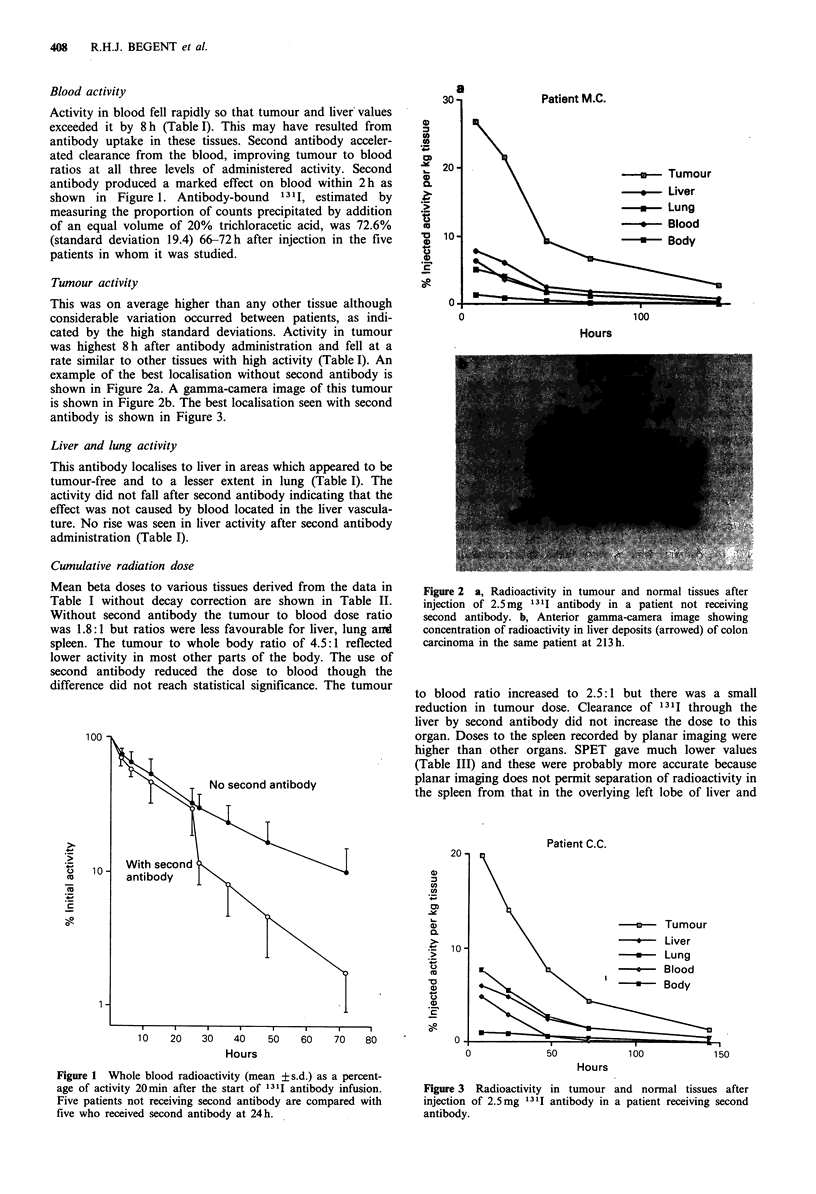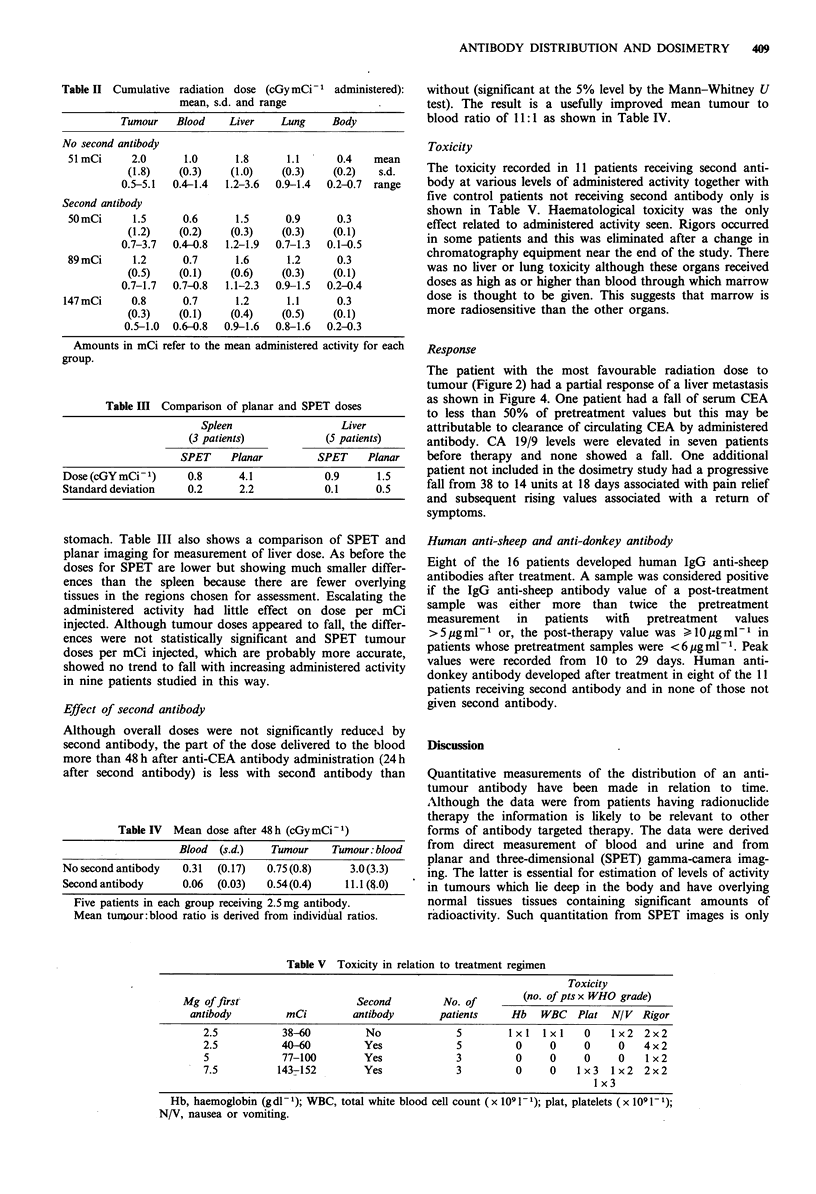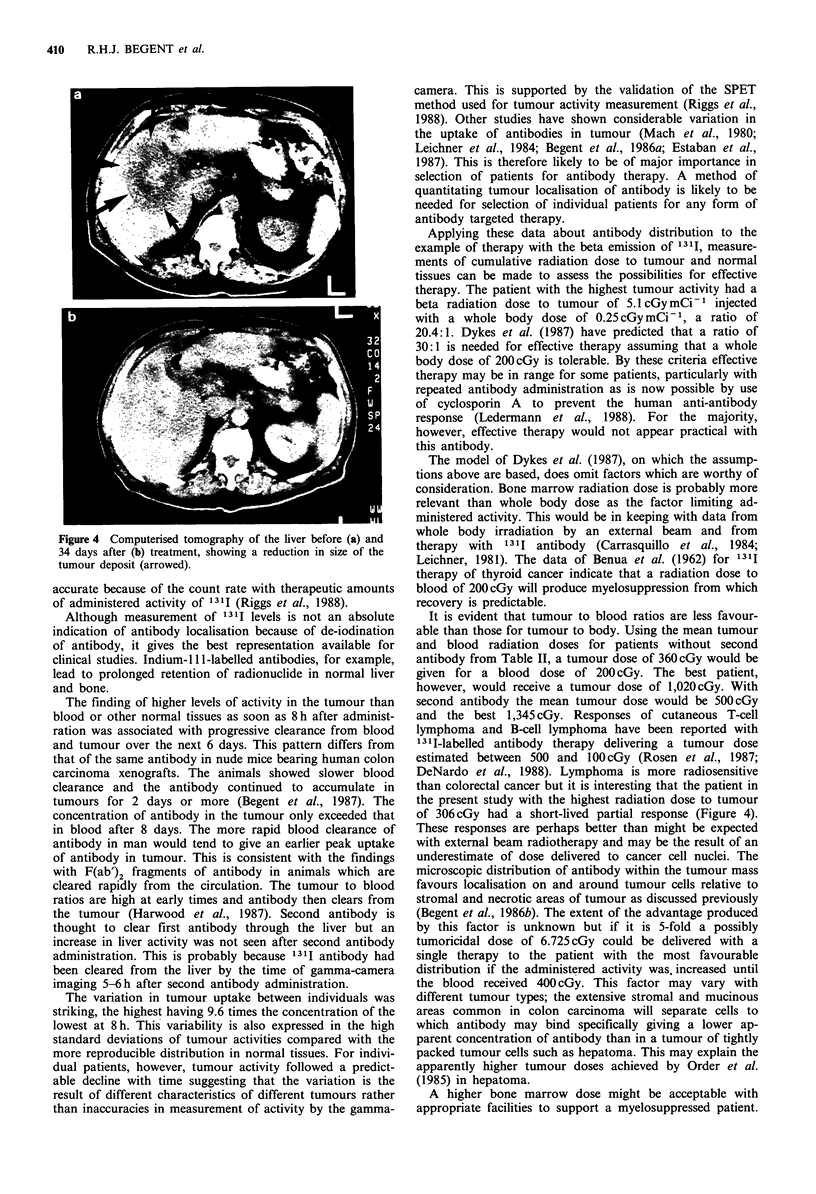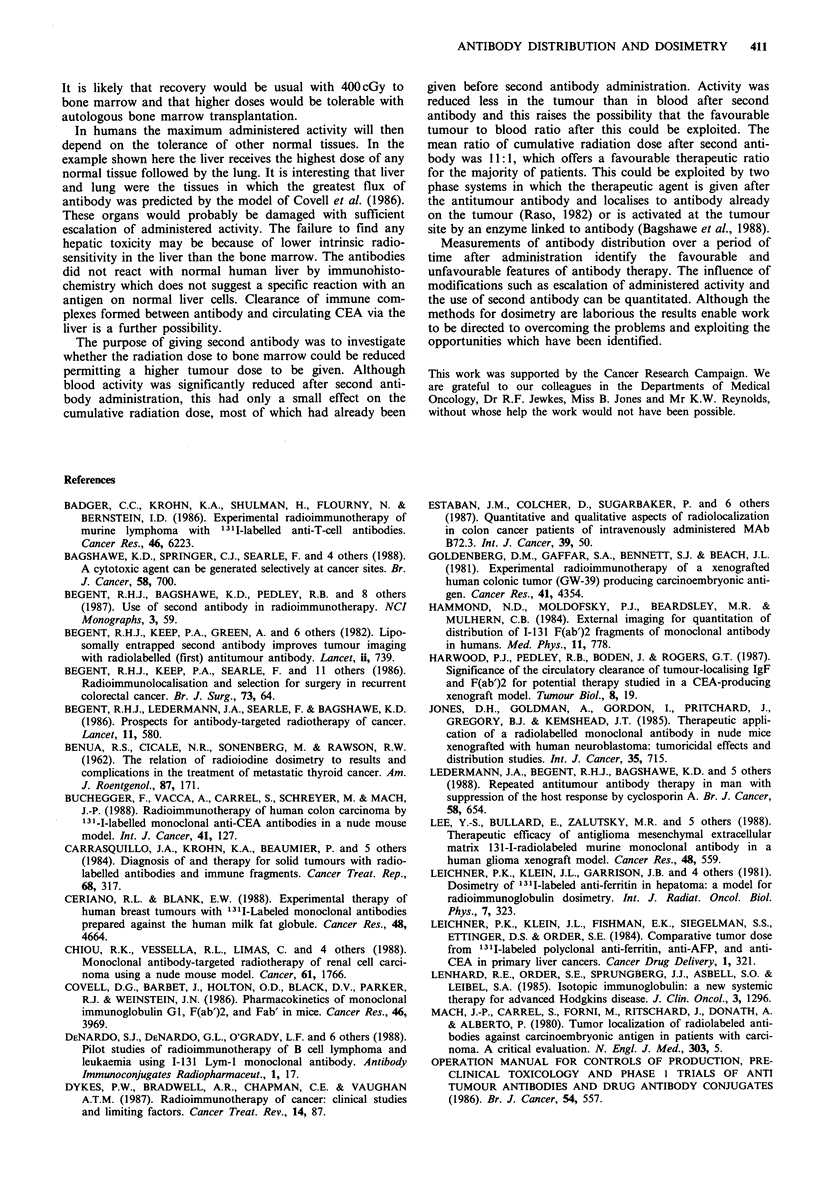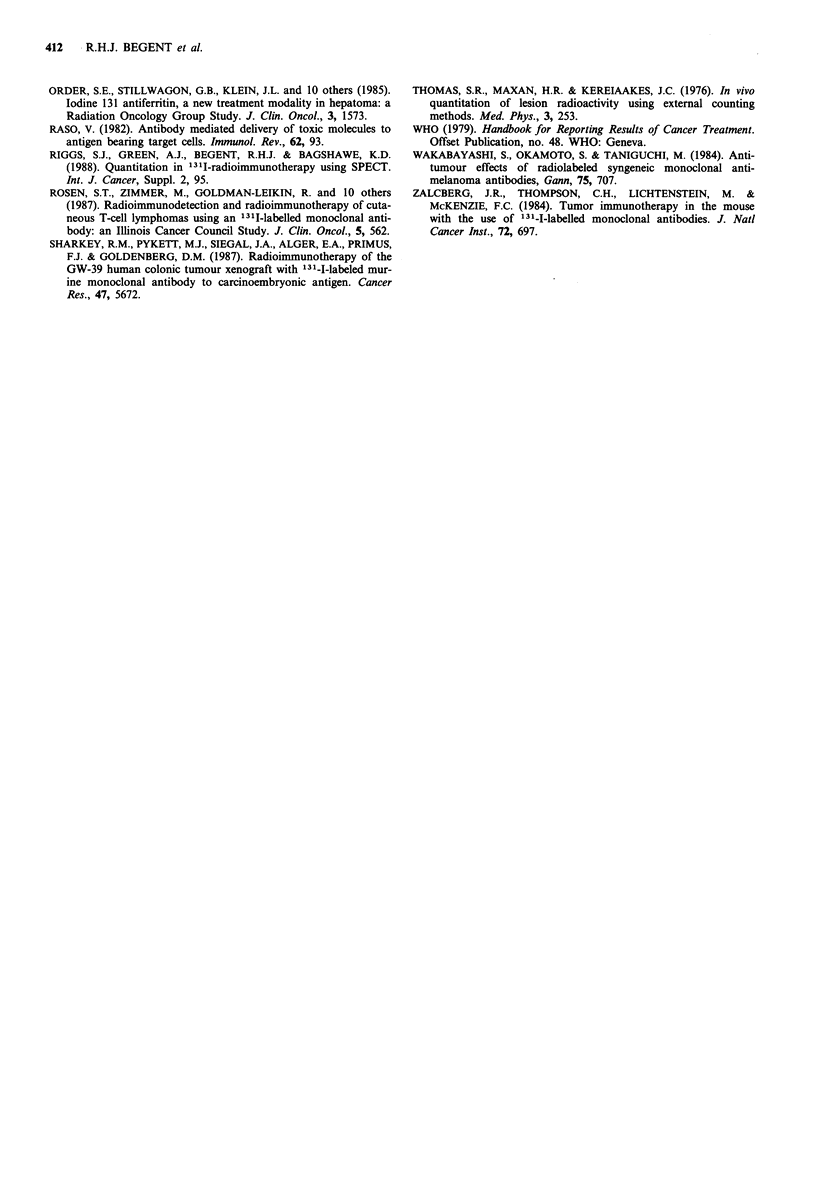# Antibody distribution and dosimetry in patients receiving radiolabelled antibody therapy for colorectal cancer.

**DOI:** 10.1038/bjc.1989.295

**Published:** 1989-09

**Authors:** R. H. Begent, J. A. Ledermann, A. J. Green, K. D. Bagshawe, S. J. Riggs, F. Searle, P. A. Keep, T. Adam, R. G. Dale, M. G. Glaser

**Affiliations:** Department of Medical Oncology, Charing Cross and Westminster Medical School, London, UK.

## Abstract

**Images:**


*Br. J. Cancer* (1989), **60**, 406–412 © The Macmillan Press Ltd., 1989

### Antibody distribution and dosimetry in patients receiving radiolabelled antibody therapy for colorectal cancer

R.H.J. Begent, J.A. Ledermann, A.J. Green, K.D. Bagshawe, S.J. Riggs, F. Searle, P.A. Keep, T. Adam, R.G. Dale & M.G. Glaser

*Cancer Research Campaign Laboratories, Department of Medical Oncology, Charing Cross and Westminster Medical School, London W6 8RF, UK.*

**Summary** The distribution of iodine-131 ($^{131}I$) labelled antibody to carcinoembryonic antigen (CEA) has been studied in 16 patients with colorectal cancer. Levels of tumour and normal tissue radioactivity were measured by serial gamma-camera imaging and counting of blood and urine. Maximum concentrations were found in tumour 8 h after administration and varied up to 9-fold in different patients. Higher levels were found on average in tumour than in any other tissue. Liver, lung and blood were the other tissues in which antibody was concentrated relative to the rest of the body. Antibody cleared from all these tissues over 1 week. Second antibody directed against the antitumour (first) antibody was given 24 h after first antibody in order to accelerate clearance from the blood. This increased the tumour to blood ratio but had little effect on other tissues. Cumulative radiation dose to tumour and normal tissue was estimated. In patients with the most efficient localisation the tumour to body ratio was 20:1 and tumour to blood ratio 5:1. This may be sufficient for effective therapy of cancer in patients selected for efficient antibody localisation. The data may be used to estimate the effect of different therapeutic strategies. For instance, in the time after second antibody administration the average tumour to blood ratio of radiation dose was 11:1, suggesting that two phase systems in which the therapeutic modality is given after a good tumour to normal tissue ratio is obtained may be effective for the majority of patients.

Antibody targeted therapy of cancer requires that a favourable distribution of antibody is sustained in tumour relative to normal tissues. Favourable distributions have been shown in mice bearing xenografts of human tumours (Sharkey *et al.*, 1987; Buchegger *et al.*, 1988; Begent *et al.*, 1987) and an antitumour effect is achieved in these systems (Goldenberg *et al.*, 1981; Sharkey *et al.*, 1987; Zalcberg *et al.*, 1984; Jones *et al.*, 1985; Buchegger *et al.*, 1988; Lee *et al.*, 1988; Wakabayashi, 1984; Chiou *et al.*, 1988; Badger *et al.*, 1986; Ceriani & Blank, 1988). Although responses are reported in patients treated systemically with $^{131}I$-labelled antitumour antibodies (Order *et al.*, 1985; Lenhard *et al.*, 1985; Carrasquillo *et al.*, 1984; Rosen *et al.*, 1987; DeNardo *et al.*, 1988) these have not been sufficient for other forms of therapy to be replaced. Understanding the reasons for this depends on a knowledge of the time course of antibody distribution in man which is lacking.

Gamma-camera imaging has given some serial measurements of radioactivity in tissues of patients receiving $^{131}I$-labelled antibody (Leichner *et al.*, 1981; Hammond *et al.*, 1984; Carrasquillo *et al.*, 1984). However, observations were made at few time points and there were no data from the first 24 h when activity may be highest. The planar imaging method used (Thomas *et al.*, 1976) is reasonably accurate when there are no overlying tissues with significant levels of activity but is unsatisfactory for measuring activity in tumours or other organs lying deep in the body.

This paper describes the use of a gamma-camera system to assess antibody distribution in tumour and normal tissues throughout the time course of therapy. Single photon emission tomography (SPET) (Riggs *et al.*, 1988) was used to give three-dimensional data for quantitation of radioactivity where planar imaging was inadequate. Patients with colorectal cancer were studied after receiving $^{131}I$ antibody to CEA and cumulative radiation doses calculated. The effect of second antibody to accelerate clearance of the antitumour antibody from the blood (Begent *et al.*, 1982, 1987) and the effect of increasing the amount of antibody administered were investigated.

Correspondence: R.H.J. Begent, Department of Medical Oncology, Charing Cross Hospital, Fulham Palace Road, London W6 8RF, UK.
Received 20 February 1989, and in revised form, 24 April 1989.

### Methods

### *Antibodies*

PK4S sheep anti-CEA antibody, affinity purified by elution from a column of CEA bound to Sepharose, has been described previously (Begent *et al.*, 1986*a*, 1987).

Antibody was radiolabelled by the chloramine T method over ice to a specific activity of 15.2–24 mCi $mg^{-1}$ antibody (median 19.4). After radiolabelling, reactivity of the antibody was confirmed by reacting it with CEA on a nitrocellulose disc. Binding was shown by autoradiography after washing the disc. Horse anti-goat or donkey anti-sheep second antibody was prepared as described previously (Begent *et al.*, 1987) and 4.0–5.3 (median 4.8) times the amount of protein of the first antibody was given. Antibodies were prepared in accordance with the Operation Manual for Control of Production, Preclinical Toxicology and Phase 1 Trials of Antitumour Antibodies and Drug Antibody Conjugates (1986).

Human anti-sheep and human anti-donkey antibody were measured by enzyme immunoassay as described by Ledermann *et al.* (1988) for human anti-mouse antibody.

### *Patients*

Patients had unresectable local recurrent or metastatic carcinoma of the colon or rectum. With ethical committee approval and after obtaining signed written consent, the following protocol was commenced.

*Day −3* Potassium iodide 180 mg t.d.s. p.o. and continued for 31 days.

*Day −1* Intradermal injection of 10 μg anti-CEA antibody and 10 μg second antibody to test for immediate type hypersensitivity. Potassium perchlorate 2000 mg q.d.f. for 5 days.

*Day 0* $^{131}I$ antibody in approximately 20 ml in a lead shielded vial was infused intravenously by displacement of the contents of the vial by 250 ml of 0.9% saline which was run through the vial. The infusion was completed in 20 min, by which time less than 5% of the initial radioactivity was detectable in the vial. Three litres of saline 0.9% alternating with dextrose 5% infused intravenously daily for 3 days to ensure rapid clearance of $^{131}I$ from the urinary tract.

*Day 1* One hour after oral administration of 600 mg soluble aspirin and 8 mg chlorpheniramine, 10% of the second antibody was given intravenously over 10 min. If there was no reaction after 20 min, the remainder of the second antibody was given over 20 min.

Toxicity to patients was measured using WHO criteria (WHO, 1979).

*Estimation of tissue activity and cumulative radiation dose*

Whole blood reactivity was measured with an LKB Compugamma in samples taken 20 min and 6 h after $^{131}I$ antibody, immediately before and 2 h after second antibody and then on days 2, 3, 4, 6, 13 and 20. Whole body activity was estimated by subtracting cumulative urine activity (LKB Compugamma) from the administered activity.

Activity in other organs was determined by gamma-camera imaging with the whole body scanning facility of the IGE Gemini gamma-camera. A high resolution 400 keV collimator with a full width half maximum (FWHM) of 11.2 mm at 100 mm in air was used for most of the work. A second 400 keV collimator with a FWHM of 14.1 mm at 100 mm in air and a sensitivity of 3 times that of the first collimator was used for imaging at later times. Before therapy an attenuation map of the patient was made with the gamma-camera above the patient couch and a flood source containing approximately 5 mCi $^{131}I$ suspended from the gamma-camera to hang beneath the patient and move with the camera. Images were taken with and without the patient in position so that attenuation of the whole body could be mapped. Opposed views of the body were then taken at approximately 6, 28, 54, 72, 144 and 216 h after antibody injection. The method of Thomas *et al.* (1976) was used to estimate organ activities at these times. In some patients serial tomographic studies were performed and activities calculated as described by Riggs *et al.* (1988). Activities in tumour lying behind other organs, such as the bladder, were calculated in this way.

Clearance curves were calculated by fitting double exponential curves to these serial estimates of blood and organ activity. Cumulated dose was calculated from integration of the clearance curves and estimates of tumour volume obtained from X-ray computerised tomography scans.

### Results

Four groups were studied: (1) five patients receiving 2.5 mg of antibody labelled with a mean of 51 mCi (range 38–60) of $^{131}I$ without second antibody; (2) five patients receiving 2.5 mg of antibody labelled with a mean of 50 mCi (range 40–60) with second antibody; (3) three patients receiving 5 mg of antibody labelled with a mean of 89 mCi (range 77–100) with second antibody; and (4) three patients receiving 7.5 mg of antibody labelled with a mean of 147 mCi (range 143–152) with second antibody. All liver and lung activities were estimated from planar imaging. Tumour activity was derived from planar images in seven cases and the remainder could only be measured by SPET because of overlying normal tissues.

*Distribution of antibody*

The time course of activity in various tissues after correction for physical decay of $^{131}I$ gives an indication of antibody distribution. Data for each of the four groups are given in Table I. Because the times of imaging varied by a few hours between patients, the values for each patient were plotted and values for the same time point read off for each patient for calculation of the mean values in Table I. The mean activity in the body is shown and levels above this represent relative concentration of antibody in the tissue concerned. Antibody concentration is seen in tumour, blood, liver and the lungs. The rest of the body had lower levels except for the spleen which occasionally showed concentration as detailed below. Activity was also seen in the urinary tract but represents $^{131}I$ separated from the antibody. No attempt was made to quantitate activity in areas of low activity where statistical error inherent in the low count rates would compromise the validity of activity measurements.

**Table I** Mean per cent injected activity per kg (s.d.) for various tissues with time, values corrected for physical decay

| *Hours* | *Tumour* | *Blood* | *Liver* | *Lung* | *Body* |
|---|---|---|---|---|---|
| *No second antibody 2.5 mg PK4S* | | | | | |
| 8 | 10.4 (11) | 6.0 (1.7) | 8.5 (2.0) | 5.8 (0.9) | 1.2 (0.3) |
| 24 | 7.0 (8.2) | 3.5 (1.2) | 6.1 (1.8) | 4.4 (1.3) | 0.9 (0.2) |
| 48 | 3.7 (3.2) | 1.7 (0.8) | 3.8 (1.9) | 2.3 (1.2) | 0.6 (0.2) |
| 72 | 2.4 (2.4) | 1.0 (0.5) | 2.4 (1.5) | 1.1 (0.1) | 0.5 (0.2) |
| 144 | 1.3 (1.2) | 0.4 (0.3) | 1.2 (0.9) | 0.5 (0.3) | |
| *Second antibody 2.5 mg PK4S* | | | | | |
| 8 | 9.0 (7.2) | 5.4 (1.5) | 7.6 (1.3) | 6.3 (1.5) | 1.1 (0.3) |
| 24 | 5.7 (4.6) | 2.9 (1.3) | 5.4 (1.3) | 4.0 (1.3) | 0.8 (0.3) |
| 48 | 3.0 (2.7) | 0.5 (0.2) | 2.7 (0.9) | 1.7 (0.9) | 0.5 (0.3) |
| 72 | 1.7 (1.4) | 0.2 (0.1) | 1.7 (0.7) | 0.8 (0.5) | 0.4 (0.2) |
| 144 | 0.6 (0.4) | | 0.8 (0.3) | 0.4 (0.1) | |
| *Second antibody 5 mg PK4S* | | | | | |
| 8 | 7.9 (5.1) | 5.4 (0.5) | 8.4 (2.9) | 6.6 (0.8) | 1.1 (0.2) |
| 24 | 4.8 (1.9) | 3.7 (0.7) | 5.7 (2.1) | 4.8 (0.7) | 0.9 (0.1) |
| 48 | 2.8 (0.7) | 0.9 (0.3) | 3.4 (1.9) | 2.6 (1.1) | 0.5 (0.1) |
| 72 | 1.6 (0.6) | 0.6 (0.2) | 2.2 (1.2) | 1.5 (0.9) | 0.4 (0.1) |
| 144 | 0.5 (0.4) | | 0.8 (0.4) | 0.5 (0.3) | |
| *Second antibody 7.5 mg PK4S* | | | | | |
| 8 | 4.7 (1.8) | 5.5 (0.8) | 7.3 (0.9) | 8.4 (0.1) | 0.9 (0.2) |
| 24 | 3.0 (0.4) | 2.4 (1.4) | 4.8 (1.3) | 5.0 (2.2) | 1.0 (0.4) |
| 48 | 1.4 (0.8) | 0.9 (0.7) | 2.3 (1.2) | 2.1 (1.6) | 0.6 (0.3) |
| 72 | 0.9 (0.5) | 0.3 (0.2) | 1.3 (0.5) | 1.0 (0.6) | 0.2 (0.2) |
| 144 | 0.4 (0.2) | | 0.6 (0.1) | 0.3 (0) | |

*Blood activity*

Activity in blood fell rapidly so that tumour and liver values exceeded it by 8 h (Table I). This may have resulted from antibody uptake in these tissues. Second antibody accelerated clearance from the blood, improving tumour to blood ratios at all three levels of administered activity. Second antibody produced a marked effect on blood within 2 h as shown in Figure 1. Antibody-bound $^{131}I$, estimated by measuring the proportion of counts precipitated by addition of an equal volume of 20% trichloracetic acid, was 72.6% (standard deviation 19.4) 66–72 h after injection in the five patients in whom it was studied.

*Tumour activity*

This was on average higher than any other tissue although considerable variation occurred between patients, as indicated by the high standard deviations. Activity in tumour was highest 8 h after antibody administration and fell at a rate similar to other tissues with high activity (Table I). An example of the best localisation without second antibody is shown in Figure 2a. A gamma-camera image of this tumour is shown in Figure 2b. The best localisation seen with second antibody is shown in Figure 3.

*Liver and lung activity*

This antibody localises to liver in areas which appeared to be tumour-free and to a lesser extent in lung (Table I). The activity did not fall after second antibody indicating that the effect was not caused by blood located in the liver vasculature. No rise was seen in liver activity after second antibody administration (Table I).

*Cumulative radiation dose*

Mean beta doses to various tissues derived from the data in Table I without decay correction are shown in Table II. Without second antibody the tumour to blood dose ratio was 1.8:1 but ratios were less favourable for liver, lung and spleen. The tumour to whole body ratio of 4.5:1 reflected lower activity in most other parts of the body. The use of second antibody reduced the dose to blood though the difference did not reach statistical significance. The tumour to blood ratio increased to 2.5:1 but there was a small reduction in tumour dose. Clearance of $^{131}I$ through the liver by second antibody did not increase the dose to this organ. Doses to the spleen recorded by planar imaging were higher than other organs. SPET gave much lower values (Table III) and these were probably more accurate because planar imaging does not permit separation of radioactivity in the spleen from that in the overlying left lobe of liver and

**Figure 2** **a**, Radioactivity in tumour and normal tissues after injection of 2.5 mg $^{131}I$ antibody in a patient not receiving second antibody. **b**, Anterior gamma-camera image showing concentration of radioactivity in liver deposits (arrowed) of colon carcinoma in the same patient at 213 h.

**Figure 1** Whole blood radioactivity (mean ±s.d.) as a percentage of activity 20 min after the start of $^{131}I$ antibody infusion. Five patients not receiving second antibody are compared with five who received second antibody at 24 h.

**Figure 3** Radioactivity in tumour and normal tissues after injection of 2.5 mg $^{131}I$ antibody in a patient receiving second antibody.

**Table II** Cumulative radiation dose (cGy mCi$^{-1}$ administered): mean, s.d. and range

| | *Tumour* | *Blood* | *Liver* | *Lung* | *Body* | |
|---|---|---|---|---|---|---|
| *No second antibody* | | | | | | |
| 51 mCi | 2.0 | 1.0 | 1.8 | 1.1 | 0.4 | mean |
| | (1.8) | (0.3) | (1.0) | (0.3) | (0.2) | s.d. |
| | 0.5–5.1 | 0.4–1.4 | 1.2–3.6 | 0.9–1.4 | 0.2–0.7 | range |
| *Second antibody* | | | | | | |
| 50 mCi | 1.5 | 0.6 | 1.5 | 0.9 | 0.3 | |
| | (1.2) | (0.2) | (0.3) | (0.3) | (0.1) | |
| | 0.7–3.7 | 0.4–0.8 | 1.2–1.9 | 0.7–1.3 | 0.1–0.5 | |
| 89 mCi | 1.2 | 0.7 | 1.6 | 1.2 | 0.3 | |
| | (0.5) | (0.1) | (0.6) | (0.3) | (0.1) | |
| | 0.7–1.7 | 0.7–0.8 | 1.1–2.3 | 0.9–1.5 | 0.2–0.4 | |
| 147 mCi | 0.8 | 0.7 | 1.2 | 1.1 | 0.3 | |
| | (0.3) | (0.1) | (0.4) | (0.5) | (0.1) | |
| | 0.5–1.0 | 0.6–0.8 | 0.9–1.6 | 0.8–1.6 | 0.2–0.3 | |

Amounts in mCi refer to the mean administered activity for each group.

**Table III** Comparison of planar and SPET doses

| | *Spleen (3 patients)* | | *Liver (5 patients)* | |
|---|---|---|---|---|
| | *SPET* | *Planar* | *SPET* | *Planar* |
| Dose (cGY mCi$^{-1}$) | 0.8 | 4.1 | 0.9 | 1.5 |
| Standard deviation | 0.2 | 2.2 | 0.1 | 0.5 |

stomach. Table III also shows a comparison of SPET and planar imaging for measurement of liver dose. As before the doses for SPET are lower but showing much smaller differences than the spleen because there are fewer overlying tissues in the regions chosen for assessment. Escalating the administered activity had little effect on dose per mCi injected. Although tumour doses appeared to fall, the differences were not statistically significant and SPET tumour doses per mCi injected, which are probably more accurate, showed no trend to fall with increasing administered activity in nine patients studied in this way.

*Effect of second antibody*

Although overall doses were not significantly reduced by second antibody, the part of the dose delivered to the blood more than 48 h after anti-CEA antibody administration (24 h after second antibody) is less with second antibody than without (significant at the 5% level by the Mann–Whitney $U$ test). The result is a usefully improved mean tumour to blood ratio of 11:1 as shown in Table IV.

**Table IV** Mean dose after 48 h (cGy mCi$^{-1}$)

| | *Blood* | *(s.d.)* | *Tumour* | *Tumour : blood* |
|---|---|---|---|---|
| No second antibody | 0.31 | (0.17) | 0.75 (0.8) | 3.0 (3.3) |
| Second antibody | 0.06 | (0.03) | 0.54 (0.4) | 11.1 (8.0) |

Five patients in each group receiving 2.5 mg antibody.
Mean tumour:blood ratio is derived from individual ratios.

*Toxicity*

The toxicity recorded in 11 patients receiving second antibody at various levels of administered activity together with five control patients not receiving second antibody only is shown in Table V. Haematological toxicity was the only effect related to administered activity seen. Rigors occurred in some patients and this was eliminated after a change in chromatography equipment near the end of the study. There was no liver or lung toxicity although these organs received doses as high as or higher than blood through which marrow dose is thought to be given. This suggests that marrow is more radiosensitive than the other organs.

*Response*

The patient with the most favourable radiation dose to tumour (Figure 2) had a partial response of a liver metastasis as shown in Figure 4. One patient had a fall of serum CEA to less than 50% of pretreatment values but this may be attributable to clearance of circulating CEA by administered antibody. CA 19/9 levels were elevated in seven patients before therapy and none showed a fall. One additional patient not included in the dosimetry study had a progressive fall from 38 to 14 units at 18 days associated with pain relief and subsequent rising values associated with a return of symptoms.

*Human anti-sheep and anti-donkey antibody*

Eight of the 16 patients developed human IgG anti-sheep antibodies after treatment. A sample was considered positive if the IgG anti-sheep antibody value of a post-treatment sample was either more than twice the pretreatment measurement in patients with pretreatment values $>5\,\mu g\,ml^{-1}$ or, the post-therapy value was $\geqslant 10\,\mu g\,ml^{-1}$ in patients whose pretreatment samples were $<6\,\mu g\,ml^{-1}$. Peak values were recorded from 10 to 29 days. Human anti-donkey antibody developed after treatment in eight of the 11 patients receiving second antibody and in none of those not given second antibody.

### Discussion

Quantitative measurements of the distribution of an anti-tumour antibody have been made in relation to time. Although the data were from patients having radionuclide therapy the information is likely to be relevant to other forms of antibody targeted therapy. The data were derived from direct measurement of blood and urine and from planar and three-dimensional (SPET) gamma-camera imaging. The latter is essential for estimation of levels of activity in tumours which lie deep in the body and have overlying normal tissues tissues containing significant amounts of radioactivity. Such quantitation from SPET images is only

**Table V** Toxicity in relation to treatment regimen

| *Mg of first antibody* | *mCi* | *Second antibody* | *No. of patients* | *Toxicity (no. of pts × WHO grade)* Hb | WBC | Plat | N/V | Rigor |
|---|---|---|---|---|---|---|---|---|
| 2.5 | 38–60 | No | 5 | 1×1 | 1×1 | 0 | 1×2 | 2×2 |
| 2.5 | 40–60 | Yes | 5 | 0 | 0 | 0 | 0 | 4×2 |
| 5 | 77–100 | Yes | 3 | 0 | 0 | 0 | 0 | 1×2 |
| 7.5 | 143–152 | Yes | 3 | 0 | 0 | 1×3<br>1×3 | 1×2 | 2×2 |

Hb, haemoglobin (g dl$^{-1}$); WBC, total white blood cell count (×10$^{9}$ l$^{-1}$); plat, platelets (×10$^{9}$ l$^{-1}$); N/V, nausea or vomiting.

**Figure 4** Computerised tomography of the liver before (**a**) and 34 days after (**b**) treatment, showing a reduction in size of the tumour deposit (arrowed).

accurate because of the count rate with therapeutic amounts of administered activity of $^{131}I$ (Riggs *et al.*, 1988).

Although measurement of $^{131}I$ levels is not an absolute indication of antibody localisation because of de-iodination of antibody, it gives the best representation available for clinical studies. Indium-111-labelled antibodies, for example, lead to prolonged retention of radionuclide in normal liver and bone.

The finding of higher levels of activity in the tumour than blood or other normal tissues as soon as 8 h after administration was associated with progressive clearance from blood and tumour over the next 6 days. This pattern differs from that of the same antibody in nude mice bearing human colon carcinoma xenografts. The animals showed slower blood clearance and the antibody continued to accumulate in tumours for 2 days or more (Begent *et al.*, 1987). The concentration of antibody in the tumour only exceeded that in blood after 8 days. The more rapid blood clearance of antibody in man would tend to give an earlier peak uptake of antibody in tumour. This is consistent with the findings with $F(ab')_2$ fragments of antibody in animals which are cleared rapidly from the circulation. The tumour to blood ratios are high at early times and antibody then clears from the tumour (Harwood *et al.*, 1987). Second antibody is thought to clear first antibody through the liver but an increase in liver activity was not seen after second antibody administration. This is probably because $^{131}I$ antibody had been cleared from the liver by the time of gamma-camera imaging 5–6 h after second antibody administration.

The variation in tumour uptake between individuals was striking, the highest having 9.6 times the concentration of the lowest at 8 h. This variability is also expressed in the high standard deviations of tumour activities compared with the more reproducible distribution in normal tissues. For individual patients, however, tumour activity followed a predictable decline with time suggesting that the variation is the result of different characteristics of different tumours rather than inaccuracies in measurement of activity by the gamma-camera. This is supported by the validation of the SPET method used for tumour activity measurement (Riggs *et al.*, 1988). Other studies have shown considerable variation in the uptake of antibodies in tumour (Mach *et al.*, 1980; Leichner *et al.*, 1984; Begent *et al.*, 1986*a*; Estaban *et al.*, 1987). This is therefore likely to be of major importance in selection of patients for antibody therapy. A method of quantitating tumour localisation of antibody is likely to be needed for selection of individual patients for any form of antibody targeted therapy.

Applying these data about antibody distribution to the example of therapy with the beta emission of $^{131}I$, measurements of cumulative radiation dose to tumour and normal tissues can be made to assess the possibilities for effective therapy. The patient with the highest tumour activity had a beta radiation dose to tumour of 5.1 cGy mCi$^{-1}$ injected with a whole body dose of 0.25 cGy mCi$^{-1}$, a ratio of 20.4:1. Dykes *et al.* (1987) have predicted that a ratio of 30:1 is needed for effective therapy assuming that a whole body dose of 200 cGy is tolerable. By these criteria effective therapy may be in range for some patients, particularly with repeated antibody administration as is now possible by use of cyclosporin A to prevent the human anti-antibody response (Ledermann *et al.*, 1988). For the majority, however, effective therapy would not appear practical with this antibody.

The model of Dykes *et al.* (1987), on which the assumptions above are based, does omit factors which are worthy of consideration. Bone marrow radiation dose is probably more relevant than whole body dose as the factor limiting administered activity. This would be in keeping with data from whole body irradiation by an external beam and from therapy with $^{131}I$ antibody (Carrasquillo *et al.*, 1984; Leichner, 1981). The data of Benua *et al.* (1962) for $^{131}I$ therapy of thyroid cancer indicate that a radiation dose to blood of 200 cGy will produce myelosuppression from which recovery is predictable.

It is evident that tumour to blood ratios are less favourable than those for tumour to body. Using the mean tumour and blood radiation doses for patients without second antibody from Table II, a tumour dose of 360 cGy would be given for a blood dose of 200 cGy. The best patient, however, would receive a tumour dose of 1,020 cGy. With second antibody the mean tumour dose would be 500 cGy and the best 1,345 cGy. Responses of cutaneous T-cell lymphoma and B-cell lymphoma have been reported with $^{131}I$-labelled antibody therapy delivering a tumour dose estimated between 500 and 100 cGy (Rosen *et al.*, 1987; DeNardo *et al.*, 1988). Lymphoma is more radiosensitive than colorectal cancer but it is interesting that the patient in the present study with the highest radiation dose to tumour of 306 cGy had a short-lived partial response (Figure 4). These responses are perhaps better than might be expected with external beam radiotherapy and may be the result of an underestimate of dose delivered to cancer cell nuclei. The microscopic distribution of antibody within the tumour mass favours localisation on and around tumour cells relative to stromal and necrotic areas of tumour as discussed previously (Begent *et al.*, 1986*b*). The extent of the advantage produced by this factor is unknown but if it is 5-fold a possibly tumoricidal dose of 6.725 cGy could be delivered with a single therapy to the patient with the most favourable distribution if the administered activity was, increased until the blood received 400 cGy. This factor may vary with different tumour types; the extensive stromal and mucinous areas common in colon carcinoma will separate cells to which antibody may bind specifically giving a lower apparent concentration of antibody than in a tumour of tightly packed tumour cells such as hepatoma. This may explain the apparently higher tumour doses achieved by Order *et al.* (1985) in hepatoma.

A higher bone marrow dose might be acceptable with appropriate facilities to support a myelosuppressed patient.

It is likely that recovery would be usual with 400 cGy to bone marrow and that higher doses would be tolerable with autologous bone marrow transplantation.

In humans the maximum administered activity will then depend on the tolerance of other normal tissues. In the example shown here the liver receives the highest dose of any normal tissue followed by the lung. It is interesting that liver and lung were the tissues in which the greatest flux of antibody was predicted by the model of Covell *et al.* (1986). These organs would probably be damaged with sufficient escalation of administered activity. The failure to find any hepatic toxicity may be because of lower intrinsic radiosensitivity in the liver than the bone marrow. The antibodies did not react with normal human liver by immunohistochemistry which does not suggest a specific reaction with an antigen on normal liver cells. Clearance of immune complexes formed between antibody and circulating CEA via the liver is a further possibility.

The purpose of giving second antibody was to investigate whether the radiation dose to bone marrow could be reduced permitting a higher tumour dose to be given. Although blood activity was significantly reduced after second antibody administration, this had only a small effect on the cumulative radiation dose, most of which had already been given before second antibody administration. Activity was reduced less in the tumour than in blood after second antibody and this raises the possibility that the favourable tumour to blood ratio after this could be exploited. The mean ratio of cumulative radiation dose after second antibody was 11:1, which offers a favourable therapeutic ratio for the majority of patients. This could be exploited by two phase systems in which the therapeutic agent is given after the antitumour antibody and localises to antibody already on the tumour (Raso, 1982) or is activated at the tumour site by an enzyme linked to antibody (Bagshawe *et al.*, 1988).

Measurements of antibody distribution over a period of time after administration identify the favourable and unfavourable features of antibody therapy. The influence of modifications such as escalation of administered activity and the use of second antibody can be quantitated. Although the methods for dosimetry are laborious the results enable work to be directed to overcoming the problems and exploiting the opportunities which have been identified.

This work was supported by the Cancer Research Campaign. We are grateful to our colleagues in the Departments of Medical Oncology, Dr R.F. Jewkes, Miss B. Jones and Mr K.W. Reynolds, without whose help the work would not have been possible.

### References

BADGER, C.C., KROHN, K.A., SHULMAN, H., FLOURNY, N. & BERNSTEIN, I.D. (1986). Experimental radioimmunotherapy of murine lymphoma with $^{131}$I-labelled anti-T-cell antibodies. *Cancer Res.*, **46**, 6223.

BAGSHAWE, K.D., SPRINGER, C.J., SEARLE, F. and 4 others (1988). A cytotoxic agent can be generated selectively at cancer sites. *Br. J. Cancer*, **58**, 700.

BEGENT, R.H.J., BAGSHAWE, K.D., PEDLEY, R.B. and 8 others (1987). Use of second antibody in radioimmunotherapy. *NCI Monographs*, **3**, 59.

BEGENT, R.H.J., KEEP, P.A., GREEN, A. and 6 others (1982). Liposomally entrapped second antibody improves tumour imaging with radiolabelled (first) antitumour antibody. *Lancet*, **ii**, 739.

BEGENT, R.H.J., KEEP, P.A., SEARLE, F. and 11 others (1986). Radioimmunolocalisation and selection for surgery in recurrent colorectal cancer. *Br. J. Surg.*, **73**, 64.

BEGENT, R.H.J., LEDERMANN, J.A., SEARLE, F. & BAGSHAWE, K.D. (1986). Prospects for antibody-targeted radiotherapy of cancer. *Lancet*, **11**, 580.

BENUA, R.S., CICALE, N.R., SONENBERG, M. & RAWSON, R.W. (1962). The relation of radioiodine dosimetry to results and complications in the treatment of metastatic thyroid cancer. *Am. J. Roentgenol.*, **87**, 171.

BUCHEGGER, F., VACCA, A., CARREL, S., SCHREYER, M. & MACH, J.-P. (1988). Radioimmunotherapy of human colon carcinoma by $^{131}$-I-labelled monoclonal anti-CEA antibodies in a nude mouse model. *Int. J. Cancer*, **41**, 127.

CARRASQUILLO, J.A., KROHN, K.A., BEAUMIER, P. and 5 others (1984). Diagnosis of and therapy for solid tumours with radiolabelled antibodies and immune fragments. *Cancer Treat. Rep.*, **68**, 317.

CERIANO, R.L. & BLANK, E.W. (1988). Experimental therapy of human breast tumours with $^{131}$I-Labeled monoclonal antibodies prepared against the human milk fat globule. *Cancer Res.*, **48**, 4664.

CHIOU, R.K., VESSELLA, R.L., LIMAS, C. and 4 others (1988). Monoclonal antibody-targeted radiotherapy of renal cell carcinoma using a nude mouse model. *Cancer*, **61**, 1766.

COVELL, D.G., BARBET, J., HOLTON, O.D., BLACK, D.V., PARKER, R.J. & WEINSTEIN, J.N. (1986). Pharmacokinetics of monoclonal immunoglobulin G1, F(ab′)2, and Fab′ in mice. *Cancer Res.*, **46**, 3969.

DENARDO, S.J., DENARDO, G.L., O'GRADY, L.F. and 6 others (1988). Pilot studies of radioimmunotherapy of B cell lymphoma and leukaemia using I-131 Lym-1 monoclonal antibody. *Antibody Immunoconjugates Radiopharmaceut.*, **1**, 17.

DYKES, P.W., BRADWELL, A.R., CHAPMAN, C.E. & VAUGHAN A.T.M. (1987). Radioimmunotherapy of cancer: clinical studies and limiting factors. *Cancer Treat. Rev.*, **14**, 87.

ESTABAN, J.M., COLCHER, D., SUGARBAKER, P. and 6 others (1987). Quantitative and qualitative aspects of radiolocalization in colon cancer patients of intravenously administered MAb B72.3. *Int. J. Cancer*, **39**, 50.

GOLDENBERG, D.M., GAFFAR, S.A., BENNETT, S.J. & BEACH, J.L. (1981). Experimental radioimmunotherapy of a xenografted human colonic tumor (GW-39) producing carcinoembryonic antigen. *Cancer Res.*, **41**, 4354.

HAMMOND, N.D., MOLDOFSKY, P.J., BEARDSLEY, M.R. & MULHERN, C.B. (1984). External imaging for quantitation of distribution of I-131 F(ab′)2 fragments of monoclonal antibody in humans. *Med. Phys.*, **11**, 778.

HARWOOD, P.J., PEDLEY, R.B., BODEN, J. & ROGERS, G.T. (1987). Significance of the circulatory clearance of tumour-localising IgF and F(ab′)2 for potential therapy studied in a CEA-producing xenograft model. *Tumour Biol.*, **8**, 19.

JONES, D.H., GOLDMAN, A., GORDON, I., PRITCHARD, J., GREGORY, B.J. & KEMSHEAD, J.T. (1985). Therapeutic application of a radiolabelled monoclonal antibody in nude mice xenografted with human neuroblastoma: tumoricidal effects and distribution studies. *Int. J. Cancer*, **35**, 715.

LEDERMANN, J.A., BEGENT, R.H.J., BAGSHAWE, K.D. and 5 others (1988). Repeated antitumour antibody therapy in man with suppression of the host response by cyclosporin A. *Br. J. Cancer*, **58**, 654.

LEE, Y.-S., BULLARD, E., ZALUTSKY, M.R. and 5 others (1988). Therapeutic efficacy of antiglioma mesenchymal extracellular matrix 131-I-radiolabeled murine monoclonal antibody in a human glioma xenograft model. *Cancer Res.*, **48**, 559.

LEICHNER, P.K., KLEIN, J.L., GARRISON, J.B. and 4 others (1981). Dosimetry of $^{131}$I-labeled anti-ferritin in hepatoma: a model for radioimmunoglobulin dosimetry. *Int. J. Radiat. Oncol. Biol. Phys.*, **7**, 323.

LEICHNER, P.K., KLEIN, J.L., FISHMAN, E.K., SIEGELMAN, S.S., ETTINGER, D.S. & ORDER, S.E. (1984). Comparative tumor dose from $^{131}$I-labeled polyclonal anti-ferritin, anti-AFP, and anti-CEA in primary liver cancers. *Cancer Drug Delivery*, **1**, 321.

LENHARD, R.E., ORDER, S.E., SPRUNGBERG, J.J., ASBELL, S.O. & LEIBEL, S.A. (1985). Isotopic immunoglobulin: a new systemic therapy for advanced Hodgkins disease. *J. Clin. Oncol.*, **3**, 1296.

MACH, J.-P., CARREL, S., FORNI, M., RITSCHARD, J., DONATH, A. & ALBERTO, P. (1980). Tumor localization of radiolabeled antibodies against carcinoembryonic antigen in patients with carcinoma. A critical evaluation. *N. Engl. J. Med.*, **303**, 5.

OPERATION MANUAL FOR CONTROLS OF PRODUCTION, PRECLINICAL TOXICOLOGY AND PHASE 1 TRIALS OF ANTI TUMOUR ANTIBODIES AND DRUG ANTIBODY CONJUGATES (1986). *Br. J. Cancer*, **54**, 557.

ORDER, S.E., STILLWAGON, G.B., KLEIN, J.L. and 10 others (1985). Iodine 131 antiferritin, a new treatment modality in hepatoma: a Radiation Oncology Group Study. *J. Clin. Oncol.*, **3**, 1573.

RASO, V. (1982). Antibody mediated delivery of toxic molecules to antigen bearing target cells. *Immunol. Rev.*, **62**, 93.

RIGGS, S.J., GREEN, A.J., BEGENT, R.H.J. & BAGSHAWE, K.D. (1988). Quantitation in $^{131}$I-radioimmunotherapy using SPECT. *Int. J. Cancer*, Suppl. 2, 95.

ROSEN, S.T., ZIMMER, M., GOLDMAN-LEIKIN, R. and 10 others (1987). Radioimmunodetection and radioimmunotherapy of cutaneous T-cell lymphomas using an $^{131}$I-labelled monoclonal antibody: an Illinois Cancer Council Study. *J. Clin. Oncol.*, **5**, 562.

SHARKEY, R.M., PYKETT, M.J., SIEGAL, J.A., ALGER, E.A., PRIMUS, F.J. & GOLDENBERG, D.M. (1987). Radioimmunotherapy of the GW-39 human colonic tumour xenograft with $^{131}$-I-labeled murine monoclonal antibody to carcinoembryonic antigen. *Cancer Res.*, **47**, 5672.

THOMAS, S.R., MAXAN, H.R. & KEREIAAKES, J.C. (1976). *In vivo* quantitation of lesion radioactivity using external counting methods. *Med. Phys.*, **3**, 253.

WHO (1979). *Handbook for Reporting Results of Cancer Treatment*. Offset Publication, no. 48. WHO: Geneva.

WAKABAYASHI, S., OKAMOTO, S. & TANIGUCHI, M. (1984). Antitumour effects of radiolabeled syngeneic monoclonal antimelanoma antibodies, *Gann*, **75**, 707.

ZALCBERG, J.R., THOMPSON, C.H., LICHTENSTEIN, M. & McKENZIE, F.C. (1984). Tumor immunotherapy in the mouse with the use of $^{131}$-I-labelled monoclonal antibodies. *J. Natl Cancer Inst.*, **72**, 697.